# A Submicrosecond-Response Ultrafast Microwave Ranging Method Based on Optically Generated Frequency-Modulated Pulses

**DOI:** 10.3390/s25010058

**Published:** 2024-12-25

**Authors:** Yifei Sun, Yongchao Chen, Longhuang Tang, Xing Jia, Heli Ma, Xiang Wang, Long Chen, Shenggang Liu, Tianjiong Tao, Jian Wu, Chengjun Li, Shuanyu Liu, Weilu Chen, Wei Gu, Jia Shi, Jidong Weng

**Affiliations:** 1 National Key Laboratory of Shock Wave and Detonation Physics, Institute of Fluid Physics, China Academy of Engineering Physics, Mianyang 622150, China; 2231070993@tiangong.edu.cn (Y.S.); ycchen16@fudan.edu.cn (Y.C.); jiaxing@caep.cn (X.J.); marcos12@126.com (H.M.); xiangwang102@126.com (X.W.); chenlongcaep@163.com (L.C.); liushenggangpla@126.com (S.L.); zjuttj@163.com (T.T.); ceuwj@zju.edu.cn (J.W.); lstrus@126.com (C.L.); 2231071001@tiangong.edu.cn (S.L.); chenwlu@mail2.sysu.edu.cn (W.C.); 17388398976@163.com (W.G.); 2 Tianjin Key Laboratory of Optoelectronic Detection Technology and System, School of Electronic and Information Engineering, Tiangong University, Tianjin 300387, China; shijia@tiangong.edu.cn

**Keywords:** displacement change, microwave FMCW radar, ultrafast ranging

## Abstract

An ultrafast microwave ranging method based on optically generated frequency-modulated microwave pulses is proposed in this study. The theoretical analysis demonstrated that nanosecond-scale linear frequency modulation microwave pulse can be obtained by femtosecond laser interference under the condition of unbalanced dispersion, which can be used to achieve a high temporal resolution of the displacement change in the measurement by the principle of frequency modulation continuous wave (FMCW) radar. The proof-of-principle experiment successfully measured the displacement change with an error of 2.5 mm and a range of 0.6 m, with a response time of 468 ns. Compared to existing microwave ranging technologies, the temporal resolution was improved by two orders of magnitude, which greatly improves the temporal resolution of distance measurement in the field of microwave FMCW radar.

## 1. Introduction

Microwave-based non-contact high temporal resolution ranging technology has attracted widespread attention over the past decade due to its high accuracy, strong anti-jamming capability, and suitability for complex environments, which has been widely applied in various fields such as aircraft navigation, structural monitoring, water level monitoring, explosive combustion monitoring, and plasma internal detection [1,2,3,4].

In the past few decades, many ranging methods have been developed, such as continuous wave (CW) interferometry [5], ultra-wideband (UWB) [6], amplitude-modulated continuous wave (AMCW) [7], and frequency-modulated continuous wave (FMCW) [8], which have been developed to attain millimeter to sub-millimeter precision in the microwave and millimeter wave frequency ranges. Among these methods, CW radar uses continuous microwaves as the carrier to interfere and demodulate the target echo signal with the transmitted signal in the time domain. By calculating the phase changes, it obtains the target’s displacement. Although this technology has measurement accuracy on the order of micrometers, its time resolution is only on the order of microseconds, and it is applicable only for relative distance measurements within the centimeter range [7,9,10,11,12]. The UWB radar measures the flight time of the same pulse ultra-wideband microwave signal and then calculates the absolute distance information. Although it can achieve absolute distance measurements with nanosecond-level time response over a range of tens of meters, its measurement accuracy is in the centimeter to millimeter range, which cannot meet the demands of precision machining [6,13,14,15,16]. The AMCW ladar uses an amplitude-modulated continuous wave signal, and the distance is accurately measured by observing the phase difference between the transmitted and received signals. Although the technology can measure a single measurement time in the order of microseconds, it can only measure relative changes in distance with micron-level accuracy, and the wavelength-limited unambiguous range is only on the order of centimeters [7]. The FMCW radar has become the fastest-growing and most widely used ranging method in microwave ranging technology based on its better balance between measurement range and accuracy. FMCW radar utilizes microwaves with a frequency that varies over time as a carrier, mixing the target echo signal with the transmitted signal and measuring the frequency and phase of the difference signal in the time domain to obtain absolute distance information. Within the measurement range of X-band to R-band, absolute distance measurement accuracy at the micrometer or sub-micrometer level is currently achievable with this technology. However, it requires high-frequency, high-bandwidth, and high-linearity swept microwave sources along with complex error correction algorithms, leading to a complicated system structure and high costs. In addition, the response time of electronically swept microwave sources is limited by the sweep rate, typically in the range of hundreds of microseconds to milliseconds, which cannot meet the high temporal resolution testing requirements for certain fast processes [17,18,19,20,21,22,23,24]. Table 1 shows a comparison of the above-described ranging methods with the approach in this paper. As can be seen from Table 1, existing ranging technologies cannot combine high temporal resolution and measurement accuracy.

This study proposes a method for ultrafast microwave distance measurement based on optically generated frequency-modulated microwave pulses, which achieves a precision of a hundred micrometers and a time resolution of a hundred nanoseconds in the X-band. A theoretical design mode of ultrafast microwave ranging composed of optically generated frequency-modulated microwave pulses was built. The principle experiment was conducted using commercially available equipment. The results denoted that this method can measure the displacement change in the meter range in the X-band with an error of 2.5 mm and a temporal resolution of about 468 ns, which is nearly two orders of magnitude smaller than the sweep time of FMCW radar.

## 2. Theoretical Analysis

### 2.1. Ranging Principle of Ultrafast Microwave

**Figure 1:**
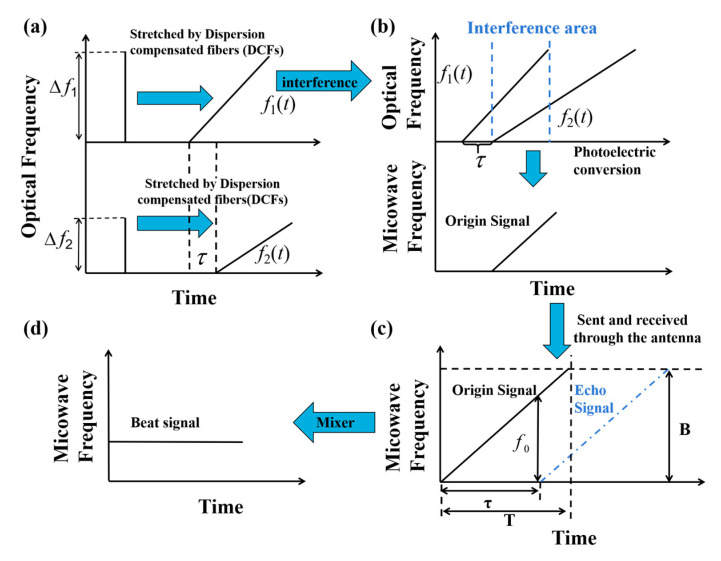
The basic principle of ultrafast microwave ranging.

The principle of ultrafast microwave ranging based on optically generated frequency-modulated microwave pulses is shown in Figure 1.

A frequency-tunable pulsed microwave waveform generator excited by a femtosecond laser is proposed in this study, which uses an unbalanced Mach-Zehnder interferometer (MZI) with frequency-to-time mapping functionality to split the light source into two arms, as shown in Figure 1a. If the pulse width of the ultrashort input optical pulse $∆t_{0}$ and the dispersion coefficient $\Phi_{i}$ of the dispersion element satisfy the condition $\left|\Delta t_{0}^{2}/2\pi \Phi_{i}\right|\ll 1$, due to the frequency-to-time mapping effect induced by the dispersion compensating fibers (DCFs), the pulses are broadened in the time domain as shown in Figure 1b, and are expressed as follows [25]:

(1) $$f_{i}(t)=C_{i}A_{i}(t)exp[j(\omega_{i}t+\frac{t^{2}}{2\Phi_{i}})]$$

where $A_{i}(t)=\{{F[x_{i}(t)]\}}_{\omega -\omega_{i}\;=\;t/\Phi_{i}}$,$x_{i}(t)$ is the Fourier transform of the optical signal filtered out of optical filters (OFs), $C_{i}$ is a constant, and $\omega_{i}$ is the central angular frequency of the optical pulse, which will affect the pulse width of the time profile.

The envelope of the output optical signal is proportional to the spectrum of the input signal. *t* is the time offset of the average delay, and the subscript *i* = 1 or 2 corresponds to the two arms, respectively, indicating the time difference between the two arms. Therefore, the time delay of the two arms is adjusted to an integer multiple of the pulse period by adjusting the VODL, which means that the optical pulses in the two arms can reach the optical coupler and interfere at the same time. The interference signal is converted by the photoelectric detector (PD), and according to the differential relationship between phase factor and frequency, the instantaneous frequency can be written as follows [26]:

(2) $$f=\frac{\left|\omega_{1}-\omega_{2}+\left(\frac{1}{\Phi_{1}}-\frac{1}{\Phi_{2}}\right)t\right|}{2\pi }$$

Based on the previous work of Hao Zhang et al. [26], the bandwidth, pulse width, and chirp rate of the generated linearly chirped microwave pulse waveform can be expressed as follows:

(3) $$B\approx \frac{c\;∆\lambda \;(1-\frac{\Phi_{2}}{\Phi_{1}})}{\lambda_{0}^{2}}$$

(4) $$T=\frac{\left|2\pi c∆\lambda \Phi_{2}\right|}{\lambda_{0}^{2}}$$

(5) $$K=\frac{B}{T}$$

where ∆λ is the relatively smaller filtering bandwidth of two OFs and $\lambda_{0}$ represents the average center wavelengths of the OFs.

Based on Equations (3) and (4), it can be concluded that the bandwidth of a linearly chirped microwave pulse is related to the amount of dispersion as well as the center wavelength and filter bandwidth of the optical filter. Since the bandwidth and center wavelength of the optical filter used in this paper are equal, the bandwidth of the linearly chirped microwave pulse is only related to the ratio of the dispersion of the two arms, and the larger the ratio, the larger the bandwidth, and the smaller the ratio, the smaller the bandwidth. In addition, the pulse width of the linearly chirped microwave pulse is related to the dispersion of one arm, and the larger the dispersion, the longer the pulse width, and the smaller the dispersion, the shorter the pulse duration.

Then, the generated linearly chirped microwave pulse is used as a microwave source and transmitted through the antenna. When the target object is relatively stationary, the reference signal and the transmitted signal produce a time delay τ (τ = 2R/*c*) as shown in Figure 1c, where R is the distance of the target object and *c* is the speed of light. The received echo signal is mixed with the transmitted reference signal to obtain the beat signal, as shown in Figure 1d. For linear frequency modulation, the beat signal carries the distance information of the target. According to the similarity triangle theorem, the distance information can be expressed as follows:

(6) $$\frac{\tau }{f_{0}}=\frac{T}{B}$$

where *T* is the pulse duration of a linearly chirped microwave pulse and *B* is the sweep bandwidth.

Equation (6) is combined with Equations (3) and (4), and the distance information can be expressed as follows:

(7) $$R=\frac{\left|\pi c\Phi_{2}\right|\;f_{0}}{\left(1-\frac{\Phi_{2}}{\Phi_{1}}\right)}$$

### 2.2. Simulation Analysis

**Figure 2:**
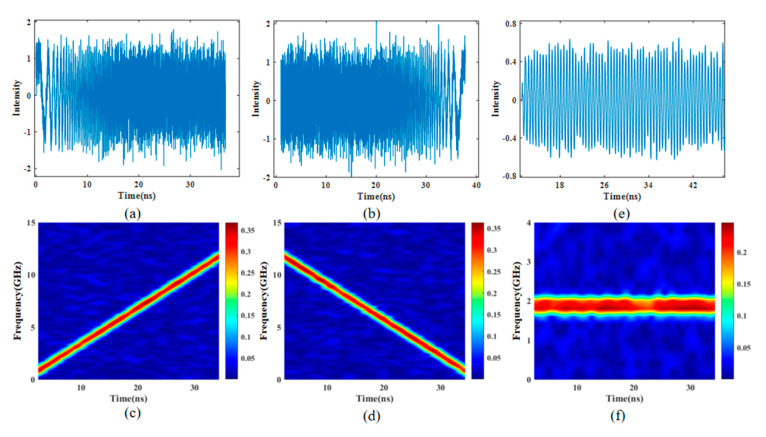
(**a**,**b**) Temporal waveforms; (**c**,**d**) STFT analyses of the simulated microwave waveforms when tuning VODLs; and (**e**,**f**) the waveform of the IF signal obtained by mixing the transmitted signal with the echo signal and its STFT analyses.

Figure 2 illustrates the numerical simulation of two types of linearly chirped microwave waveforms and their short-time Fourier transform (STFT) analysis, as well as the intermediate frequency (IF) signal obtained from the beat frequency between the transmitted and echo signals, including its waveform and STFT. Random noise with a noise figure of 0.3 is added to the ideal waveforms to simulate the real waveforms. The filtering bandwidths in Figure 2a,b are the same, but the time delay difference between the two arms is 952 ps. Both waveforms are centered at 6.75 GHz, with a maximum instantaneous frequency of 12 GHz and a pulse duration of 35 ns. So the chirp rate is 0.34 GHz/ns. By adjusting the time delay, the frequency modulation directions can be made opposite. Therefore, the waveform in Figure 2a is an up-chirp from 0 to 12 GHz (see Figure 2c), while the waveform in Figure 2b is a down-chirp from 12 GHz to 0 GHz (see Figure 2d–f), which shows the waveform and short-time Fourier transform (STFT) analysis of the intermediate frequency (IF) signal obtained by mixing the transmitted signal with the echo after the linearly chirped microwave pulse is reflected by the object. Random noise with a noise figure of 0.3 is added to the ideal waveform. The target distance is assumed to be 40 cm. As shown in Figure 2f, the frequency value of the IF signal is around 1.9 GHz, which is consistent with the theoretical calculation. As can be seen from Equation (6), the measurement range is related to the pulse width of the linearly chirped microwave pulse, and the longer the pulse duration, the longer the measurement distance. It should be noted that the repetition frequency of the femtosecond laser used in this paper is 21.38 MHz, so the maximum pulse width of the linearly chirped microwave pulse is 46.77 ns. If the pulse width is too long, it leads to overlapping waveforms, which affect the accuracy of subsequent displacement measurements. The theoretical distance resolution of this research can be calculated by $\Delta R=\frac{c}{2B}(\frac{M1}{M2})$, where M1 represents the number of sample points and M2 stands for FFT points. The number of sampling points in this research is 1600, and the number of points of FFT transformation is 72,727; therefore, the theoretical distance resolution of the method is 1.1 mm.

## 3. Experimental Setup

**Figure 3:**
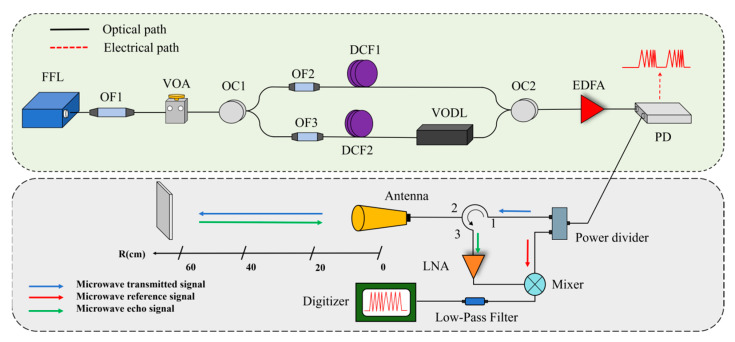
Schematic of the proposed method.

The schematic diagram of the submicrosecond-response ultrafast microwave ranging method based on optically generated frequency-modulated pulses is shown in Figure 3. The optical source used in our experiment is a femtosecond fiber laser (FFL), of which the repetition frequency, output power, and center wavelength were 21.38 MHz, 22.30 mW, and 1550 nm, respectively. The spectrum of the femtosecond laser was first shaped into a rectangular profile through a bandpass optical filter (OF1, 1550 nm ± 4 nm), which then passes through a variable attenuator to match the optical coupled power from both arms. The 50:50 optical coupler (OC1) splits the optical source into two arms. Two dispersion-compensated fibers (DCFs), DCF1 and DCF2, with dispersion values of −8391 ps^2^ and −8624 ps^2^ were used to introduce different chirps into the optical pulses through the wavelength–time mapping effect. Their spectra are formed by two optical filters, OF1 and OF2 (OF1/OF2, 1550 ± 4 nm), with approximately rectangular profiles. Variable optical delay lines (VODLs) are added to the second arm to adjust the difference in the time delay between the two arms caused by the two DCFs to an integer multiple of the pulse period. The optical signals from both arms are coupled through a 50:50 optical coupler (OC2), amplified by an erbium-doped fiber amplifier (EDFA), and then converted into a linearly chirped microwave waveform by a photodetector (PD). The resulting linearly chirped microwave pulse is used as the microwave source. Through a power divider (operating frequency of 6–18 GHz, isolation ≥18 dB, and insertion loss ≤0.8 dB), the microwave signal is divided into two parts, which were used as the transmitted signal and the reference signal, respectively. The antenna (gain of 18 dB and VSWR of 1.5) is connected to a microwave circulator (operating frequency of 7–12.4 GHz, insertion loss of 1.2 dB, and isolation of 40 dB) for transmission and reception of the transmitted signal and its echo signal at the same time. After the 3 ports of the circulator, the power of the echo signal is increased by a low-noise amplifier. Finally, the echo signal and the reference signal are mixed by a mixer (RF/LO working frequency of 10–44 GHz, IF working frequency of DC −14 GHz, and conversion loss of 7.5 dB), and the difference frequency signal is recorded by a digitizer.

## 4. Results and Discussion

**Figure 4:**
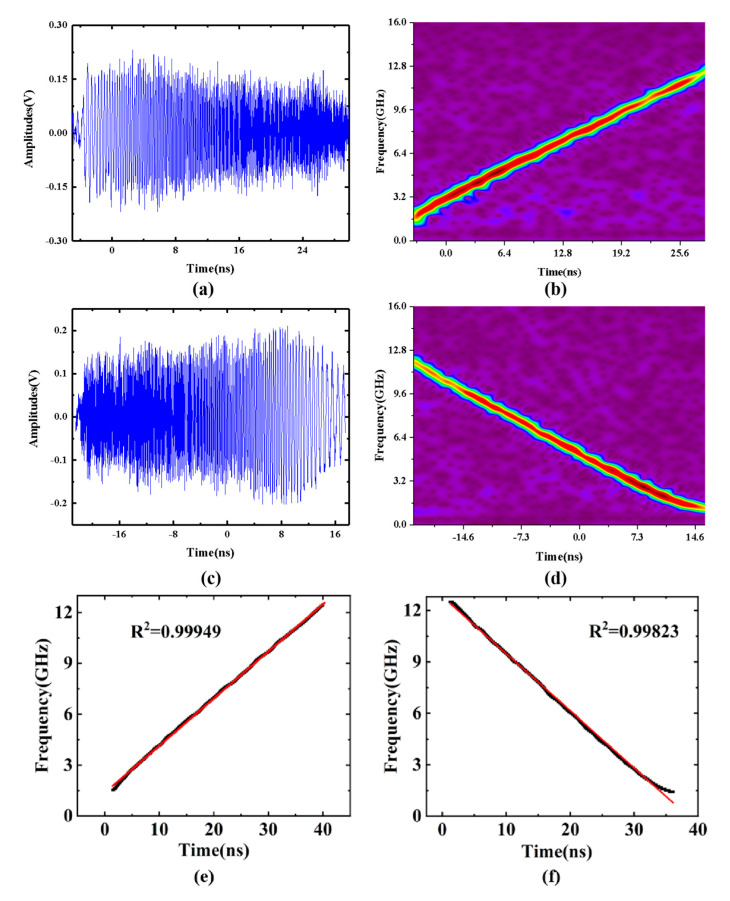
(**a**,**c**) Temporal waveforms; (**b**,**d**) STFT analyses of the generated microwave waveforms; and (**e**,**f**) the linearity of the up-chirp and down-chirp waveforms.

Figure 4 illustrates two distinct types of microwave waveforms captured at the photodetector (PD) output using a high-speed real-time oscilloscope and their short-time Fourier transform (STFT) analysis. Figure 4a displays the waveform when the time delay difference between the two optical paths is precisely tuned to an integer multiple of the pulse period. The measured sweep bandwidth is approximately 10.7 GHz, ranging from 1.3 GHz to 12 GHz, with the maximum instantaneous frequency observed around 12 GHz. The linear chirp microwave pulse does not start from 0 GHz due to the presence of a low-pass filter (frequency range: 1.3–18.0 GHz). The pulse duration is approximately 35 ns. When the time delay is adjusted to 950 ps, the ends of the stretched optical pulse signals in the optical frequency domain are aligned, and the resulting down-chirp waveforms are shown in Figure 4c,d. As shown in Figure 4b,d, the instantaneous frequencies at the middle time are located at 6 GHz, with the chirp directions being opposite. The sweep rates (K) are nearly identical for both types of waveforms. The measured waveforms maintain an approximately rectangular shape but exhibit slight amplitude variations. These variations may be attributed to unpredictable noise from the PD and fluctuations in the polarization states of the two optical paths. Figure 4e,f illustrate the linearity of the short-time Fourier transform for the up-chirp and down-chirp waveforms. The linearity of the up-chirp is 0.99949, and the linearity of the down-chirp is 0.99823. The main reason for the poor linearity is the nonlinear effect caused by the DCFs.

**Figure 5:**
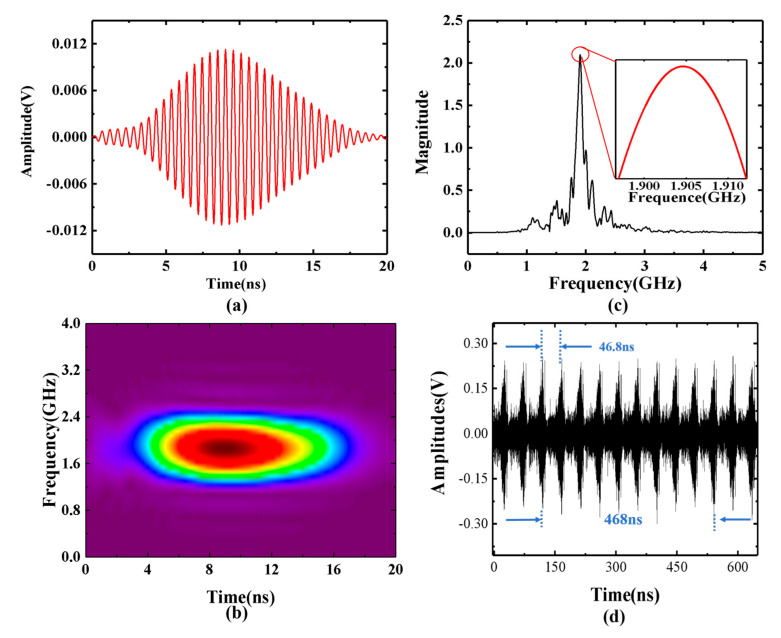
(**a**) Temporal waveforms; (**b**) STFT analyses of the IF signal; (**c**) typical Fourier transform analysis spectrum; and (**d**) generated microwave pulses.

Figure 5 shows the results of ultrafast microwave ranging based on optically generated frequency modulation pulses. Firstly, we place the target object close to the antenna port and take the average value of multiple measurements as the zero point (R = 0 cm). Then the distance information can be obtained by subtracting the zero point result from each measured distance result. Then, the target is placed at a distance of 40 cm from the antenna. The IF signal waveform is recorded using an oscilloscope. According to Equation (7), the measured distance can be calculated using the Fourier transform result of the intermediate frequency signal waveform captured by the oscilloscope as shown in Figure 5a–c, which illustrate the IF signal waveform obtained from the transmitted and echo signals after beat frequency processing, along with its short-time Fourier transform (STFT) analysis. The frequency of the IF signal is approximately 1.9 GHz, which is consistent with the previous simulation results. Figure 5c shows a typical Fourier transform spectrum, in which the zero padding operation is performed before the Fourier transform of the signal to increase the number of points of the FFT transform to improve the frequency resolution. After the Fourier transform, there is an obvious Gaussian peak. We use all points on the half peak for Gaussian fitting. After fitting, the maximum value point is obtained as our peak frequency. The peak frequency is at 1.891 GHz, corresponding to a measured distance of 40.732 cm. The spectrum also reveals three low-amplitude side lobes adjacent to the main peak, which can be attributed to the absence of microwave-absorbing materials designed to suppress multipath reflections. Figure 5d shows the train of generated microwave pulses, which indicates that the time interval between two contiguous pulses is about 46.8 ns. Taking 10 pulse sequences as the single measurement time, the single measurement time is 468 ns.

**Figure 6:**
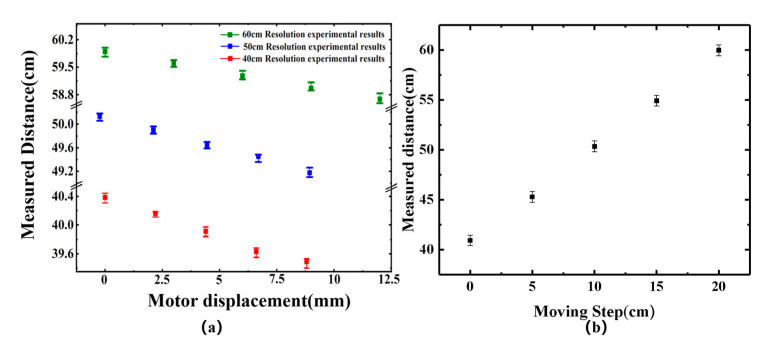
(**a**) The ranging resolution at distances of 40 cm, 50 cm, and 60 cm. (**b**) Ranging results.

In order to further study the ranging resolution of the system, a comparative experiment of microwave ranging based on optically generated frequency modulation and a motorized control displacement platform was established. In the experiment, the target is fixed on an electric displacement platform. The minimum increment of motion of the motorized stage is 5 μm/step. The accuracy of the motorized displacement control stage is in the few microns. Figure 6a shows the resolution error bars at 40 cm, 50 cm, and 60 cm. The experimental results show that the system achieves a ranging resolution of 3 mm. Figure 6b illustrates the results of microwave ranging based on optical frequency modulation. In the experiment, the target position is adjusted every 5 cm by a motorized displacement control platform, and its accuracy is in the order of a few microns. For each target position, repeated distance measurements were taken 50 times. The average of every 50 measurements was calculated to obtain the measurement result. For distances of 40 cm, 45 cm, 50 cm, 55 cm, and 60 cm, the standard deviations were measured as 526 µm, 542 µm, 541 µm, 519 µm, and 541 µm, respectively, and the difference between the maximum and minimum values was measured as 2.29 mm, 2.25 mm, 2.22 mm, 2.35 mm, and 2.57 mm, respectively. In addition, the measurement results were 40.938 cm, 45.698 cm, 50.472 cm, 55.232 cm, and 59.981 cm, respectively. Therefore, taking the motorized displacement platform as a reference, the displacement accuracy is ± 2.5 mm.

**Figure 7:**
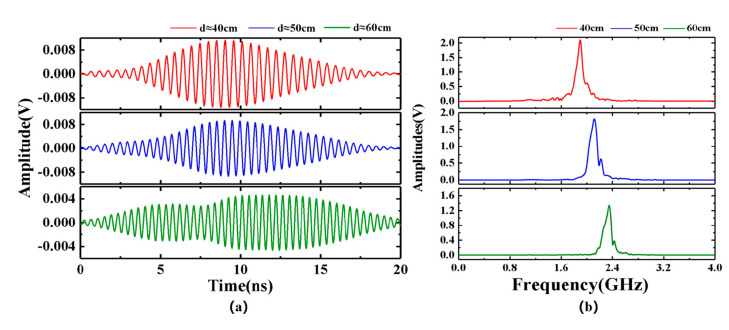
(**a**) Waveform diagram of an IF signal in the distance range of 40, 50, and 60 cm. (**b**) Fourier transforms of IF signals at distances of 40, 50 and 60 cm.

As shown in Figure 7, the waveforms and their Fourier transform results at different distances were recorded, specifically at approximately 40 cm, 50 cm, and 60 cm. It is evident that as the measurement distance continues to increase, the frequency of the intermediate frequency (IF) signal exhibits a gradual upward trend, which aligns with our previous theoretical analysis. In addition to the change in frequency, the power of the beat signal decreases continuously with increasing measurement distance. This reduction in power directly results in a corresponding decrease in the waveform amplitude. A small bulge on the right side of the dominant frequency signal in Figure 7b can be attributed to the absence of microwave-absorbing materials used to suppress multipath reflection.

**Figure 8:**
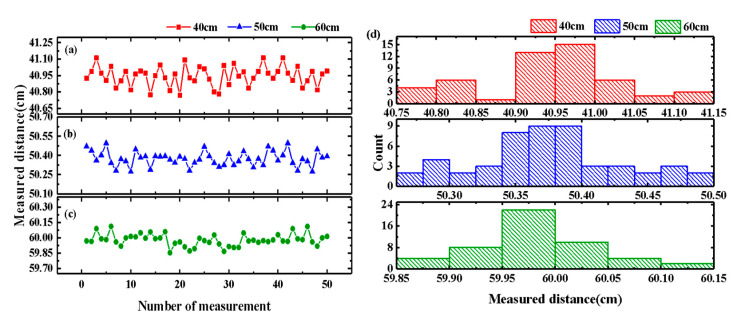
The measured distance stability of 50 measurements at about (**a**) 40 cm, (**b**) 50 cm, and (**c**) 60 cm. (**d**) The distribution histogram of measured values at different distances.

To better assess the measurement stability across various distance ranges, distance measurements were performed 50 times at each range to facilitate statistical analysis. The results of repeated measurements across three distance ranges are illustrated in Figure 8a–c. The standard deviations for the measured distances of 40 cm, 50 cm, and 60 cm were determined to be 526 µm, 541 µm, and 545 µm, respectively. Since the standard deviation σ is directly related to the measurement uncertainty $u_{a}$, the 95% confidence interval as an example was used, $u_{a}=2\sigma /\sqrt{N}$, where *N* represents the number of measurements. The distance values were expressed as the measured mean ± the uncertainty, which was 40.938 cm ± 149 µm, 50.472 cm ± 154 µm, and 59.981 cm ± 154 µm, corresponding to the 95% confidence interval. It is evident that, although the standard deviation of the measured distance increases with the extending measuring range, the measurement stability remains consistently around 500 μm. The distribution characteristics of the measured values at three different distances were statistically analyzed. The results, presented in the histogram in Figure 8d, demonstrate that the measured distance values exhibit a general tendency towards a normal distribution. It can be inferred that boosting the transmitted microwave power to improve the signal-to-noise ratio of the difference frequency signals may further enhance the measurement stability for distant targets.

## 5. Conclusions

In summary, this study presents a submicrosecond-response ultrafast microwave ranging method based on optically generated frequency-modulated microwave pulses. This method combines unbalanced dispersion and spectral filtering with an easy-to-operate microwave ranging technique that relies on frequency modulation continuous wave (FMCW) radar. Theoretical analysis indicates that by adjusting the filter bandwidths, dispersion, and time delays of the two arms, the desired linear chirp microwave pulses can be generated for detected signals in the ranging results. Additionally, the distance information can be obtained by analyzing the beat frequency of the echo signal and the reference signal. Proof-of-principle experiment results show that the method can use a frequency-modulated microwave pulse with a bandwidth of 3 GHz (limited by the bandwidth of the microwave device) for ultrafast microwave ranging. The standard deviations at measurement distances of 40 cm, 50 cm, and 60 cm are 526 µm, 541 µm, and 545 µm, respectively, with a pulse repetition frequency of 2.133 MHz. The resolution is 3 mm. The measured error is 2.5 mm. This method is suitable for complex engineering environments and is expected to play an important role in a wider range of precise microwave ranging applications.

## Figures and Tables

**Table 1:** The microwave ranging method proposed in this paper compared with the existing technology.

Microwave Ranging Technology	Measured Distance and Displacement	Single Measurement Time	Measurement Error
CW radar ranging technology	1 m 10 μm	Not mentioned	12.5 mm
UWB radar ranging technology	>10 m Not mentioned	Nanosecond level	3.07 mm
AMCW radar ranging technology	0.4 m 2 mm	1.6 μs	8 μm
FMCW radar ranging technology	1.4 m 1 mm	1 ms	5 μm (500 times on average)
This paper	0.6 m 0.1 m	468 ns	2.5 mm

## Data Availability

Data are contained within the article.
